# The effectiveness of a tablet-based video game that stimulates cognitive, emotional, and social skills in developing academic skills among preschoolers: study protocol for a randomized controlled trial

**DOI:** 10.1186/s13063-022-06875-9

**Published:** 2022-11-09

**Authors:** Cristian A. Rojas-Barahona, Jorge Gaete, Mauricio Véliz, Ramón D. Castillo, Saray Ramírez, Ricardo Araya

**Affiliations:** 1 Faculty of Psychology, Research Center on Cognitive Sciences, Universidad de Talca, Talca, Chile; 2 Research Center for Students Mental Health (ISME), Faculty of Education, Universidad de los Andes, Santiago, Chile; 3 Millennium Nucleus to Improve the Mental Health of Adolescents and Youths, Millennium Science Initiative, Santiago, Chile; 4 Facultad de Ciencias de la Educación, Universidad de Talca, Talca, Chile; 5 Department of Health Service & Population Research, King’s College London, London, UK

**Keywords:** Cognitive skills, Emotional skills, Academic skills, Tablet play, Preschools, Randomized controlled trial

## Abstract

**Background:**

Evidence suggests that children from low-income families begin the preschool stage with less academic and non-academic skills development compared to higher-income families. There are several successful experiences of early stimulation of cognitive and social-emotional skills; however, there is scarce evidence of the effectiveness of a video game that incorporates the stimulation of these skills simultaneously. This study aims to evaluate the effectiveness of a video game in stimulating cognitive, emotional, and social competence skills in developing academic skills in socioeconomically disadvantaged preschool children.

**Methods:**

A cluster-randomized controlled trial design will be used. A tablet-based video game that stimulates cognitive and socio-emotional skills to improve the development of academic skills is compared with a tablet-based game where students draw and paint with no explicit stimulation of cognitive and socio-emotional skills. Eighteen schools and 750 Chilean preschool students will be recruited. The effectiveness of the intervention will be assessed using a direct evaluation of children on literacy learning and pre-calculation skills at baseline, immediately after stimulation, and at 6, 12, 18, and 24 months post-intervention. The mediating effect of working memory, inhibitory control, emotion recognition, and prosocial behaviours will be assessed on the effectiveness of the intervention.

**Discussion:**

The proposed study will be the first to test the effectiveness of a tablet-based video game stimulating cognitive and social-emotional skills to improve academic skills in socioeconomically disadvantaged preschool children in Chile, controlling for gender, age (in months), mental health, and baseline conditions of stimulated skills.

**Trial registration:**

ClinicalTrials.gov NCT05224700. Registered on February 2022

## Administrative information

Note: the numbers in curly brackets in this protocol refer to SPIRIT checklist item numbers. The order of the items has been modified to group similar items (see http://www.equator-network.org/reporting-guidelines/spirit-2013-statement-defining-standard-protocol-items-for-clinical-trials/).

**Table:** 

Title {1}	The effectiveness of a tablet-based video game that stimulates cognitive, emotional, and social skills on developing academic skills among preschoolers: study protocol for a randomized controlled trial.
Trial registration {2a and 2b}.	Trial identifier NCT05224700 in Clinical Trials [ClinicalTrials.gov] under the registry name: “Early Development of Academic Skills in the Classroom: Short- and Mid-term Effects of a Tablet-based Game That Stimulates Cognitive, Emotional, and Social Skills for Pre-kindergarten Children”; [registered in February 2022]
Protocol version {3}	Version 1 of 01-25-2022
Funding {4}	This research is funded by the National Research and Development Agency [ANID]. Unique ID: Fondecyt Regular 1210989; and by ANID – Millennium Science Initiative Program – NCS2021_081.
Author details {5a}	C. Rojas: Faculty of Psychology, Research Center on Cognitive Sciences, Universidad de Talca-Chile. J. Gaete: Research Center for Students Mental Health (ISME), Faculty of Education, Universidad de los Andes. Chile. Millennium Nucleus to Improve the Mental Health of Adolescents and Youths, Millennium Science Initiative, Chile. M. Veliz: Facultad de Ciencias de la Educación, Universidad de Talca-Chile. R. Castillo: Faculty of Psychology, Research Center on Cognitive Sciences, Universidad de Talca-Chile. S. Ramírez: Research Center for Students Mental Health (ISME), Faculty of Education, Universidad de los Andes. Chile. Millennium Nucleus to Improve the Mental Health of Adolescents and Youths, Millennium Science Initiative, Chile. R. Araya: Department of Health Service & Population Research, King's College London, London, United
Name and contact information for the trial sponsor {5b}	Investigator initiated clinical trial; Cristian Rojas (Principal Investigator). Contact information: E-mail: c.rojas@utalca.cl; Phone +71 2201747; Address: Avenida Lircay S/N, Campus Lircay, Faculty of Psychology, Universidad de Talca, Maule Region.
Role of sponsor {5c}	Funders played no role in the design of the study, collection, management, analysis, and interpretation of the report. They will not have ultimate authority over any of these activities.

## Introduction

### Background and rationale {6a}

Several authors [1] have expressed that improving learning and socialization skills has many advantages in the prevention of behaviour problems, the quality of life of children, and long-term health and economic outcomes. Evidence suggests that children from low-income families may begin the preschool stage with lower development of academic skills (such as precalculus and initial literacy) and non-academic skills (such as emotional and social competence), when compared to children from higher-income families [2]. Such differences could increase long-term social-emotional, educational, and health disparities [3]. Between the ages of 3 and 7, critical aspects of human development happen, such as emotion recognition, control of behaviour [4], and the development of Executive Functions (EF) [5]. Therefore, it becomes essential to stimulate these skills among socially vulnerable children.

The EFs are integrated by different components participating in goal-directed behaviour, where inhibitory response, attention, and working memory seem critical [6]. In recent years, the development of EFs has been a significant focus of research, among other reasons, because [6] (a) there is growing evidence that EFs are crucial to school success; (b) EFs are deficient in several learning/developmental disorders; (c) children in socioeconomically disadvantaged context seem to have lower EF development before entering formal education when compared when their counterparts from more affluent families; and (d) there is increasing evidence that EFs can be stimulated in children with typical and atypical development.

Within EFs, working memory (WM) and inhibitory control (IC) have been identified as critical components in the development of academic skills such as early literacy and precalculus [7], in addition to the evidence that they can be stimulated early in development [8]. WM and IC can be developed if they are stimulated directly; however, it is less clear, especially for WM, the impact of their stimulation on the development of other skills such as literacy and precalculus [9–11]. Studies have also found the relationship between EFs and other behavioural and non-cognitive skills [12], such as social [13] and emotional development [14].

Emotional understanding refers to the fashion children identify, predict, and explain emotions regarding themselves and others. Around 3 years of age, children can start labelling emotional expressions. Then, they rapidly continue to advance in the development of emotion recognition. Rosnay et al. [15] have reported that children who are more successful in understanding emotional cues are more likely to develop social skills and prosocial responses to their peers and to form positive interpersonal relationships that encourage adaptation to social situations. With regard specifically to social ability and behaviour involving the other, it becomes necessary to divide components into those inherent to the person, with those that are dyadic. We will dwell on the precursor elements of a personal level, such as the ability to identify and monitor related behaviours, closely linked to emotional and executive functions [16]. In this sense, several studies have observed that the development early in the life of early emotional and social competencies facilitates the successful interactions of children with others and favours better academic achievements and mental health [17, 18].

In general, interventions that stimulate emotional and social skills include varied strategies and practices that are usually not limited to promoting a particular skill. Therefore, it is difficult to isolate the effect of the stimulation of a specific skill. A meta-analysis by Blewitt et al. [19], which included the revision of programmes for children between 2 and 6 years old, found that children exposed to social and emotional stimulation interventions showed a significant improvement in social and emotional competencies, learning, behavioural regulation, and decreased their emotional and behavioural problems. Most of the revised interventions have been studied in developed countries, are complex, sustained in time, and require a lot of resources in personnel and material that are not readily available in vulnerable socio-economic contexts.

In recent years, there has been a growing use of devices with touch screens by children. It has been observed that their use in an educational context for academic purposes has favourable evidence [20], especially when these applications have been created with careful designs and content selection [21]. A recent systematic review by Griffith et al. [22] concludes that there is evidence that interactive applications can be helpful and accessible tools to support early academic development. Herodotou [23] found that most of the research involving technological devices evaluated cognitive aspects and, in general, neglected the socio-emotional aspects. In addition, of the studies analysed, which report results in various areas of children’s development, eight present findings in the development of academic skills, evidencing advances that guide the contribution of devices with touch screens in these skills, that is, in learning.

Finally, there are several successful experiences of early stimulation of cognitive skills using technological devices, such as COGMED for children aged 7–17 years with ADHD by Klingberg et al. [24], and Memory Booster for children aged 5 to 8 years of St Clair-Thompson and collaborators [8]. Other interventions promoting socio-emotional skills using interactive teaching strategies have also accumulated good evidence, such as PATHS created by Greenberg and collaborators [25] and Incredible Years by Carolyn Webster-Stratton [26]. However, we did not find an evaluation of the effectiveness of a programme that incorporates the stimulation of cognitive and social-emotional skills using an interactive video game on a tablet for preschool children, followed up in the medium term.

## Objectives {7}

The main objective of this study is to assess the effectiveness of a programme of stimulating cognitive skills (working memory and inhibitory control), emotional skills (recognition of emotions), and social competence (choice of prosocial behaviours), in the development of academic skills of initial literacy and precalculus in socioeconomically disadvantaged preschool children. The outcomes will be assessed immediately after the intervention, at 6, 12, 18, and 24 months post-intervention, controlling for gender, age (in months), mental health, and baseline conditions of the stimulated skills.

The specific objectives are (a) to determine the acceptability and ease of use of the programme of stimulation of cognitive, emotional, and social competence skills in socioeconomically disadvantaged preschool children; (b) to evaluate the changes in the variables working memory, inhibitory control, emotion recognition, and the choice of prosocial behaviours, in both groups (intervention and comparison group), immediately after the intervention and at 6, 12, 18, and 24 months post-intervention; (c) to determine the effectiveness of the programme of stimulation of cognitive, emotional, and social competence skills in the development of the academic ability of early literacy and in the development of the academic skill of precalculus in socioeconomically disadvantaged preschool children, immediately after the intervention and at 6, 12, 18, and 24 months post-intervention, controlling for gender, age (in months), mental health, and baseline conditions of the stimulated skills; and (d) to explore the influence of potential mediators (working memory, inhibitory control, emotion recognition, and choice of prosocial behaviours) on the observed effects of the cognitive, emotional, and social competence skill stimulation programme, controlling for gender, age (in months), mental health, and basal conditions of the stimulated skills.

We hypothesize that the programme will have high acceptability among socioeconomically disadvantaged preschool children. Immediately after the end of the intervention, there will be an improvement in the variables working memory, inhibitory control, recognition of emotions and choice of prosocial behaviours, with greater development in the intervention group, which will be maintained throughout the different measurements. The same will happen in developing early literacy and precalculus academic skills. The effect of the programme is mediated by working memory, inhibitory control, emotion recognition, and choice of prosocial behaviours, controlling for gender, age (in months), mental health, and baseline conditions of stimulated skills.

## Trial design {8}

**Fig. 1:**
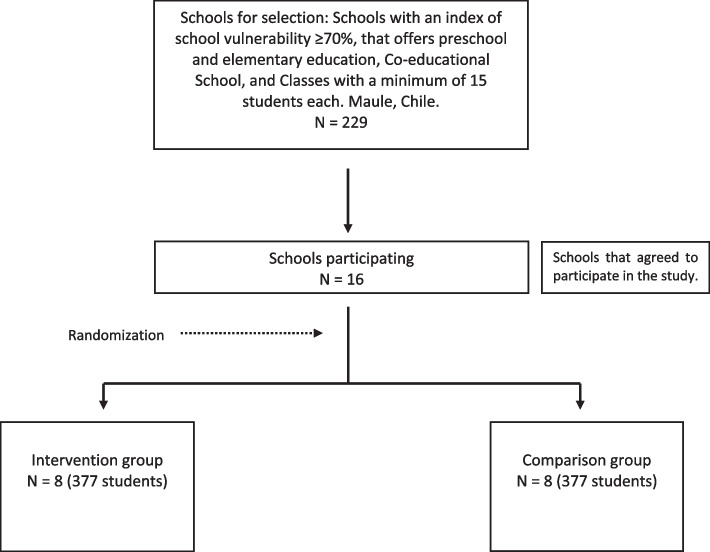
Flowchart

This is a cluster randomized controlled trial, parallel-group type, where the tablet-based video game stimulating cognitive, emotional, and social skills is compared to a tablet-based video game involving only painting. The effectiveness of this programme will be evaluated by one pre-stimulation measurement, and five post-stimulation measures, immediately after stimulation, at 6, 12, 18, and 24 months. The patient allocation is randomized with a ratio of 1:1. See Fig. 1.

## Methods: participants, interventions, and outcomes

### Study setting {9}

This study will be carried out in schools with preschool education located in two cities in the Maule region (Talca and Linares), Chile.

### Eligibility criteria {10}

#### Inclusion criteria

- Establishments with an index of school vulnerability (ISV) ≥70% (based on the information provided by participating families)
- Establishment that offers preschool and elementary education
- Establishment located in the region of Maule, Chile
- Mixed-sex schools.
- Classes with a minimum of 15 students each

#### Exclusion criteria

- School is implementing a similar programme.
- Special education school.
- Children with a reported cognitive disability.

### Who will take informed consent? {26a}

All schools in Region del Maule will be assessed for eligibility. The eligibility of schools to participate in this study will be evaluated based on the abovementioned criteria. After the schools have been considered eligible, all eligible schools will be invited. Then, the principal of the selected schools will receive the study information. After school officials agree to participate in the study and sign an authorization form, preschool educators and pre-kindergarten assistants will be invited to participate (in conjunction with kindergarten and first-grade educators as part of the longitudinal study). Subsequently, the parents or guardians of the students will be informed about the study in a meeting and invited to participate. Along with this information, parents will receive the informed consent form and must sign it if they agree to have their children participate in the study. Finally, all authorized children will be asked for their willingness to participate in the study. Even if you have the consent of children, if the child does not confirm that they want to participate, they will not be forced.

### Additional consent provisions for collection and use of participant data and biological specimens {26b}

No samples were collected. There are no plans for future studies using data collected in this trial.

### Interventions

#### Explanation for the choice of comparators (6b)

The control group (active) will not work with the programme of stimulation of cognitive skills and socio-emotional; instead, this group will use a tablet-based game where they must paint during the 20 sessions of 30 min, two times a week. At the end of the activity, you will be given a sticker for your participation. In this group, the educators will have the same training in cognitive, emotional and social competence skills as those in the intervention group. They will also be trained in using the game to paint, and meetings will be held every 15 days to evaluate possible doubts. This way, an attempt to avoid the effects of the programme dependent on the educators is made since, in both groups, a similar treatment would be received. The knowledge will remain within the educational institution for future work in the area. Like the intervention group, the group will be guided by the kindergarten educator responsible for the course and will work with the entire course group simultaneously (each child will have an individual tablet with headphones). All children who wish to play will be able to participate (to avoid discrimination); however, only children who have parental/guardian consent and the consent of the child themselves will be evaluated. A research team member will be present at each session to ensure that the session flows correctly.

#### Intervention description {11a}

The intervention group will use the tablet-based video game to stimulate cognitive and emotional skills and social competence. The intervention will be implemented through a tablet game programme that promotes cognitive skills (working memory and inhibitory control), emotional (recognition of emotions), and social competence (choice of prosocial behaviours) individually in the classroom context. This programme will be held during the academic year during school hours and has 20 sessions of 30 min, two times a week (in total two and a half months), where students will be in groups in the room, but each child will work with a tablet with headphones individually. At the end of the activity, each child will receive a sticker for participation. All activities are games with the following structure: an introduction with instructions and selection of the protagonist (avatar: which can be modified according to hair colour, skin, sex, and clothes, who speaks directly to the child), followed by a practice exercise to make sure that the children understand what they should do (it is repeated until the child performs the routine correctly), and finally, the stimulation activity (4 different activities with five exercises each). In the first ten sessions, working memory and emotion recognition are stimulated together (4 activities for each skill in each session). In the remaining ten sessions, inhibitory control and the choice of prosocial behaviours are stimulated.

#### Criteria for discontinuing or modifying allocated interventions {11b}

The students of the intervention and comparison group will participate in all the programme sessions; however, the data of the children with cognitive disabilities (with previous diagnosis) will not be considered for the analyses. Children with cognitive disabilities, in the context of this study, are those children who have been identified by a trained school professional indicating that they cannot clearly understand the instructions of the instruments. The main reasons for excluding these children from the analyses are that the measurement tools are not validated for children with cognitive disabilities. These children may have difficulty understanding the instructions making the results less reliable.

In addition, while students cannot leave the classroom if they do not want to participate in the sessions, they can leave the study at any time and for any reason, if they wish, without any consequences. If children leave the study, their information and collected data will not be analysed.

#### Strategies to improve adherence to interventions {11c}

A record of the children’s attendance and percentage of participation in each session (for both groups) will be made. In addition, a research team member will register each session’s incidents and observations. For example, there will be a registry of the number of children playing every 5 min, and the reasons why the children are not performing the task will also be noted (e.g. going to the bathroom, a problem with the tablet, etc.). Finally, once every 15 days, a meeting will be held with all the educators to evaluate the possible doubts and difficulties in developing the programme.

#### Relevant concomitant care permitted or prohibited during the trial {11d}

The schools participating in the study cannot implement other programmes stimulating cognitive and socio-emotional skills during the trial.

#### Provisions for post-trial care {30}

There is no harm nor potential harm in this trial. The intervention group will receive an intervention built under the best evidence available to stimulate preschoolers’ cognitive, social, and emotional skills. The active control group will receive a tablet game programme where they will only paint. We have included several benefits for children and schools participating in the study: (1) A report with the data collected will be provided to all schools (without the children’s identity) to help the institutions plan future measures to be included in the regular teaching activities to stimulate development. (2) Additionally, those parents or main caregivers who request information about the results of the measures carried out on their children will be granted. (3) We have offered to all schools some training opportunities for early educators to gain new skills related to promoting cognitive and non-cognitive skills among children. This training will be implemented after the final follow-up assessment.

### Outcomes {12}

#### Primary outcomes

1. Tests of academic skills:
  1.1. Initial literacy learning: Two tests will be used: Texas Lee, designed by the Texas Education Agency [27] in collaboration with the University of Houston to evaluate reading in Spanish-speaking children. This instrument has been validated for Chile by Medina et al. [28]. It considers the following dimensions: Knowledge of the printed, Knowledge of the alphabet, Phonological/phonemic awareness, and Oral comprehension (*a* =.82). To evaluate vocabulary, the Spanish adaptation of the Kaufman Assessment Battery for Children (K-ABC) [29] will be used, and it was also validated for this age group by Medina et al. [28] (*a* =.79). Each correct answer will be registered as one point. The total score means the number of correct answers. We will assess the difference between groups at each point in time (immediately after stimulation, at 6, 12, 18, and 24 months), using the mean score of the group, adjusting by baseline mean scores.
  1.2. Pre-calculation skills: Woodcock-Muñoz (Battery III [30]), includes the following subtests of the achievement test: Quantification, Verbal Counting, Identification of numbers, and Applied Problems. Split-half reliability for Spanish-speaking children ages 4 and 5 ranges from .93 to .95. Each correct answer will be registered as one point. The total score means the number of correct answers. We will assess the difference between groups at each point in time (immediately after stimulation, at 6, 12, 18, and 24 months), using the mean score of the group, adjusting by baseline mean scores.

#### Secondary outcomes

2. Mental health problems: Goodman’s “Strengths and Difficulties Questionnaire” (SDQ) [31] will be utilized. It consists of a series of emotional and behavioural problems experienced by children between 4 and 16 years old. It has four subscales of psychological difficulties (of 5 items each): emotional symptoms, behavioural problems, hyperactivity/attention problems, peer problems, and prosocial behaviour. These subscales can be combined into a Difficulties subscale with a total score. The version for children and young people in Chile was validated by the team of this project Gaete et al. [32], with an internal consistency through the McDonald’s Omega index, which fluctuates between .76 and .85 (report from main caregivers). We will assess the difference between groups at each point in time (immediately after stimulation, at 6, 12, 18, and 24 months), using the mean score of the group, adjusting by baseline mean scores.
3. Cognitive Skills Tests:
  3.1. Working memory: 2 memory tests will be applied: visual-spatial and phonological. For the visuospatial area, the “Corsi Cube test” [33] will be administered. It involves repeating a sequence of up to nine spatially separated identical blocks on a screen. The series starts simple but becomes more complex until the subject’s performance decreases (internal consistency between .75 and .78. For the phonological area, the auditive memory subtest of Woodcock-Muñoz (Battery III [30]) (internal consistency .80) will be used. A sequence of audio messages is presented from lowest to greatest difficulty, and the child is asked to remember these messages. Each correct answer will be registered as one point. The total score means the number of correct answers. We will assess the difference between groups at each point in time (immediately after stimulation, at 6, 12, 18, and 24 months), using the mean score of the group, adjusting by baseline mean scores.
  3.2. Inhibitory control: the “Hearts & Flowers” task [34] will be used, which is a hybrid that combines elements of Simon and spatial tasks of Stroop and contains congruent and incongruous essays. For congruent trials, subjects must obey the rule: “Press on the same side as the stimulus (Hearts)”. For incongruous trials, subjects should follow the rule: “Press on the side opposite the stimulus (Flowers)”. The test showed a Cronbach alpha of .83. Each correct answer will be registered as one point. The total score means the number of correct answers. We will assess the difference between groups at each point in time (immediately after stimulation, at 6, 12, 18, and 24 months), using the mean score of the group, adjusting by baseline mean scores.
4. Socio-emotional Skills Tests:
  4.1. Emotion recognition: the “Assessment of Children’s Emotional Skills” (ACES) by Schultz et al. [35] will be administered. The test comprises three subscales: facial expressions, social situations, and social behaviour. The current project will administer the subscale of facial expressions (*a* =.68). Each child will need to identify correctly 16 facial expressions. Each correct answer will be registered as one point. The total score means the number of correct answers. We will assess the difference between groups at each point in time (immediately after stimulation, at 6, 12, 18, and 24 months), using the mean score of the group, adjusting by baseline mean scores.
  4.2. Selection of prosocial behaviours: the “Challenging Situations Task” (CST) by Denham et al. [36] will be employed, reporting a Cronbach alpha of .83. The instrument assesses children’s ability to solve problems. Children are presented with peer problems, and their responses are classified into four possibilities: prosocial, aggressive, crying, and avoidant. Each option selected by children will be summed in a subsequent subscale. The prosocial subscale will be used as the main comparator between groups. We will assess the difference between groups at each point in time (immediately after stimulation, at 6, 12, 18, and 24 months), using the mean score of the group, adjusting by baseline mean scores.

### Participant timeline {13}

Table 1 shows the participant’s timeline.

**Table 1:**
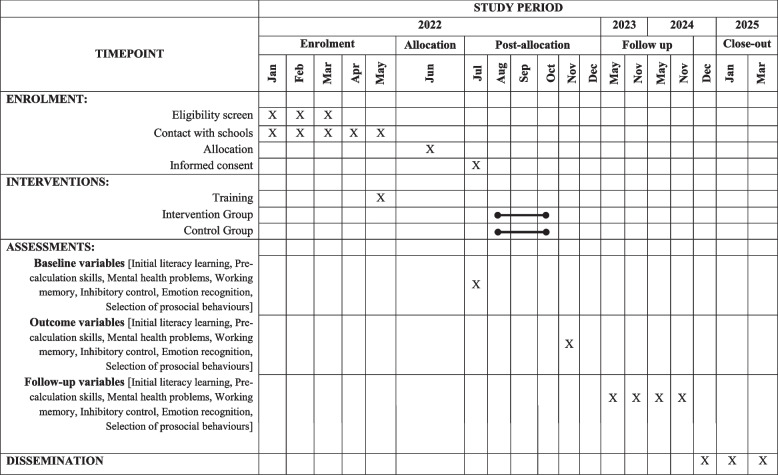
Standard Protocol Items: Recommendations for Interventional Trials (SPIRIT) diagram

### Sample size {14}

To determine a significant difference (alpha =0.05) in the measure of the primary outcome (academic skills: early literacy/precalculus), between the comparison and intervention groups, with a power of 80%, we need to recruit six schools with a total of 264 students per group, with an average of 44 students per school (distributed in two courses). For the sample calculation, the Stata statistical package was used, and the “clustersampsi” command, which indicates the differences between groups concerning an average score and considers the correlation effect between the subjects within the same school through the intracluster correlation coefficient (ICC = 0.02) [11]. The information to feed this equation was extracted from a previous study [37], where the control group in the early literacy variable post-intervention measure had a score of 20.06 (DS = 8.32). Assuming a difference in favour of the intervention group of 0.4 DS, the sample size was calculated for this study. In addition, it is estimated that 10% of eligible students might not want to participate, and estimating a 20% loss in follow-up, each group should initially recruit 377 students. This implies that four extra schools will be recruited per group, totalling 16.

### Recruitment {15}

The research team already has good collaboration networks with municipalities and schools in Chile. Strategies to achieve adequate school enrolment to achieve the target sample size will consist of contacting and presenting to the municipal study authorities who are expected to help establish contact with school authorities. In addition, research assistants will communicate directly and inform school authorities about the study’s purpose, requirements and duration.

## Assignment of interventions: allocation {16}

### Sequence generation {16a}

Schools will be randomly distributed to either group with an allocation ratio of 1:1 through an automated procedure using Excel (Microsoft) and the command “RAND”. A statistician independent of the research team will perform the randomization.

### Concealment mechanism {16b}

After the randomization and allocation, schools will be informed by a research assistant of the group belonging by email and confirmed by telephone. Each school will only receive their information of allocation. Additionally, this information will not be disclosed to the assessment research team (outcome evaluators) to keep them blind to the schools’ allocation.

### Implementation {16c}

An independent statistician will perform the randomization to assign to the study arms, and this statistician will give this information to a research assistant. Later, the research assistant will inform schools by email and confirm by telephone.

## Assignment of interventions: blinding

### Who will be blinded {17a}

This is a double-blinded trial, blinded to the outcome evaluators and the data analyst. Outcome evaluators will not be informed about the group of belonging and will be instructed not to ask the students or the school authorities about the school's condition. A data analyst will work with the final dataset, where the group condition will be masked.

### Procedure for unblinding if needed {17b}

For the nature of the intervention, the participants will know the allocation of the school.

## Data collection and management

### Plans for assessment and collection of outcomes {18a}

Each participant will be evaluated individually on six occasions, one before the intervention and five after the intervention, using the tasks focused on working memory, inhibitory control, emotion recognition, choice of prosocial behaviours and precalculus skills and early literacy, in 2 sessions lasting approximately 40 minutes each (with breaks in each). Trained psychologists will conduct the children’s assessment in a 1:1 ratio. Each trained psychologist will arrange the evaluation time during school hours, which will occur in a pre-determined location on the school premises. The conditions for the evaluation will be ensured: privacy, no distractions, and safety. The trained psychologist will be responsible for the well-being of the children in the assessment, and a supervisor may be in place or on call for any support if needed. To ensure data quality recollection, evaluators will fulfil a daily report of incidents with the children, and a supervisor will solve the problems. In addition, the supervisor will make field visits to check if these assessments follow the evaluation protocol. On the other hand, the parents will answer a mental health questionnaire of the child during the periods of the children’s assessments. The instruments to be used in the measurements are described below.

### Plans to promote participant retention and complete follow-up {18b}

To ensure greater rigour in the research design, a record of the children’s attendance and percentage of participation of the children in each session will be made (for both groups: intervention and comparison). An observation record will be kept, made by a member of the research team, of each session carried out, where the number of children who are playing will be recorded every 5 min, and the reasons why the children are not performing the task will be noted (e.g. went to the bathroom, a problem with the tablet). In addition, the observation protocol will include multiple-choice items that will try to capture the main difficulties encountered during the programme implementation and how the educator and the assistant are responsible for supporting the children during the session to carry out the activity. Students will not have access to the software between sessions.

### Data management {19}

After the participants have completed the questionnaires, the data will be entered into a secure platform without identifying information (each participant will be assigned an encrypted ID number). The original copies of the instruments will be filed and stored, under lock and key, in a self-storage, along with the list linking the participants’ names and ID numbers. Only the principal investigator, research assistants in charge of data entry, and the statistician will have access to the database.

### Confidentiality {27}

Research data will be stored using a study identification code for each participant. The key to the identification code list will only be available to the research team mentioned above. It will be documented and safeguarded according to research guidelines after the completion of the study. No participant identification details will be reported in publications or any study report.

### Plans for collection, laboratory evaluation, and storage of biological specimens for genetic or molecular analysis in this trial/future use {33}

Not applicable. There are no biological samples in this assay.

## Statistical methods

### Statistical methods for primary and secondary outcomes {20a}

First, descriptive analyses will be performed to evaluate the balance of variables between groups at the beginning of the study. The primary analysis will be conducted based on intention-to-treat. This analysis includes all randomized subjects in the groups, regardless of whether or not they complete the treatment or follow-up period. Multivariate regression models of mixed effects will be carried out considering as a dependent variable the performance in the tests of academic skills (initial literacy and precalculus) and as an independent variable belonging to the intervention or comparison group, controlling for gender, age (in months), mental health, and performance in the same tests in the baseline evaluation. Regression models will consider the nested nature of the data (clustering), since students’ performance within the same educational establishment tends to be correlated. While primary outcomes will be considered as those obtained after the intervention, analyses will be repeated for follow-up at 6, 12, 18, and 24 months. This will allow for evaluation of the maintenance of the effect of the intervention until first grade. We will use mixed-effect regression models of repeated measures to assess convergence and divergence between groups in follow-up times. Subgroup analyses will be performed for the primary outcome variable using interaction terms in the regression models between the randomized groups and the gender variable. This will allow us to evaluate the moderating effect. Sensitivity analyses will also be performed to assess the impact of lost data using multiple imputations. The results with and without imputed data will be presented and compared. The tests will be done with Stata 15.0.

Finally, multilevel models of mediation will be adjusted using Mplus 6, entering all the hypothesized mediators simultaneously: working memory, inhibitory control, recognition of emotions, and choice of prosocial behaviours. We will calculate the effect of the intervention on each mediator, the effect of each mediator on the primary outcome, and the effect of the intervention that each mediator mediated. For each model, we will calculate the intervention’s indirect effects and the unique indirect effect for each mediator. Then, we will calculate the direct effect of the programme. Because randomization will be at the school level, we will understand the school as second-level and individuals as first-level. To control variability across schools, we will use the Mplus stratification option. Then, we will separate the models for initial literacy and prequel skills. All models will be adjusted for gender, mental health, and values of the mediating variables in the baseline evaluation. Covariations between all variables within time will be allowed.

### Interim analyses {21b}

Not applicable. There will not be interim analyses because the data will be analysed at the end of the trial.

### Methods for additional analyses (e.g. subgroup analyses) {20b}

Subgroup analyses will be performed to explore differences between males and females. The statistical approach will follow the procedures described above.

### Methods in analysis to handle protocol non-adherence and any statistical methods to handle missing data {20c}

The primary outcome will be assessed using an intention-to-treat analysis. Every participant randomized to the trial will enter the primary analysis. Accordingly, participants who drop out prematurely, or are non-compliant with the study intervention, will be included in the primary analysis within the respective group they have been assigned to at randomization. Multiple imputations will be used to handle missing data in the primary and secondary analyses.

### Plans to give access to the full protocol, participant-level data, and statistical code {31c}

The datasets produced during the current study will be available in an international database repository called UK Data Service.

## Oversight and monitoring

### Composition of the coordinating centre and trial steering committee {5d}

This is a monocentre study designed, performed, and coordinated at the University of Talca, Chile. Daily support for the trial is provided by the principal investigator, who supervises the trial. Additionally, a data manager organizes data collection and assures data quality. The study coordinator helped in trial registration and will coordinate study visits and reports. Study research assistants will help to identify potential participating schools, collect informed consent, ensure follow-up according to protocol, and resolve doubts from school staff or participants.

The study team will meet weekly during the whole duration of the study. There is no trial steering committee or stakeholder and public involvement group. The Ethical Scientific Committee of the University of Talca will check the presence and completeness of the investigation.

### Composition of the data monitoring committee, its role, and reporting structure {21a}

A monitor from the Ethical Scientific Committee of the University of Talca will check at least twice during the investigation of the presence and completeness of the research. This committee is independent of the sponsor and has no competing interests; further details about its charter can be asked via email: cec@utalca.cl.

### Adverse event reporting and harms {22}

The intervention or the data collection procedure does not infer harm among the participants. However, any situation that compromises participants’ physical and psychological integrity during all different actions related to the project will be registered. Additionally, any incident of discomfort or harm reported by the child or educator will be recorded through a form that a research assistant will fill out, both in the evaluations and in the intervention. This information will be managed by the Principal Investigator and Project Coordinator and reported to the Ethical Scientific Committee, and, if necessary, they will contact school authorities and main caregivers. Any adverse events will be reported in the trial publications.

### Frequency and plans for auditing trial conduct {23}

A monitor from the Ethical Scientific Committee of the University of Talca will check at least twice during the project the presence and completeness of the investigation files, such as informed consents, inclusion and exclusion criteria, and data collection and storage.

### Plans for communicating important protocol amendments to relevant parties (e.g. trial participants, ethical committees) {25}

All substantial amendments will be notified to the ethics committee of the University of Talca. If amendments concern or affect participants, they will be informed about the changes. If needed, additional consent will be requested and registered. Also, online trial registries will be updated accordingly.

## Dissemination plans {31a}

The results of this research will be entirely disclosed in international peer-reviewed journals. Both positive and negative results will be reported. A summary of the results will be given to school authorities.

## Discussion

It is known that there are several successful experiences of early stimulation of cognitive skills and social-emotional skills. However, we did not find an evaluation of the effectiveness of a programme that incorporates both skills together, even more so using tablets and for preschool children, followed up in the medium term. With the proposed study, he hopes there will be evidence for the first time of a tablet-based cognitive and social-emotional skills stimulation programme that improves academic skills in socioeconomically disadvantaged preschool children, controlling for gender, age (in months), mental health, and baseline conditions of the stimulated skills.

There are some potential limitations to keep in mind. There would be an unknown impact of the COVID-19 pandemic on preschool students because the pandemic and its implemented public health measures have drastically changed the way children could interact socially with their peers. There is also a risk that the implementation will be lost due to potential school closures due to COVID-19 infections.

## Trial status

Recruitment will begin in April–June 2022. The current protocol is version 1 of April 2022. It is estimated that patient recruitment will be completed around July–August 2022. All items included in this protocol can be found on Clinical Trial Id NCT05224700 [ClinicalTrials.gov].

## Data Availability

The final trial dataset will be available in an international data repository called UK Data Service.

## Abbreviations

EF: Executive functions
WM: Working memory
IC: Inhibitory control
ACES: Assessment of Children’s Emotions Skills
CST: Challenging Situations Task
SDQ: Strengths and Difficulties Questionnaire
K-ABC: Kaufman Assessment Battery for Children
